# Seipin—still a mysterious protein?

**DOI:** 10.3389/fcell.2023.1112954

**Published:** 2023-02-03

**Authors:** Veijo T. Salo

**Affiliations:** Structural and Computational Biology Unit, European Molecular Biology Laboratory (EMBL), Heidelberg, Germany

**Keywords:** seipin, lipid droplet, lipid droplet-ER contact sites, membrane contact site, endoplasmic reticulum, mitochondria-ER contact sites

## Abstract

Cells store excess energy in the form of lipid droplets (LDs), a specialized sub-compartment of the endoplasmic reticulum (ER) network. The lipodystrophy protein seipin is a key player in LD biogenesis and ER-LD contact site maintenance. Recent structural and *in silico* studies have started to shed light on the molecular function of seipin as a LD nucleator in early LD biogenesis, whilst new cell biological work implies a role for seipin in ER-mitochondria contact sites and calcium metabolism. In this minireview, I discuss recent insights into the molecular function of seipin.

## 1 Introduction

Seipin mutations have been linked to severe congenital lipodystrophy (BSCL2), motor neuronal disorders and congenital encephalopathy (Magré et al., 2001; Windpassinger et al., 2004; Ito and Suzuki, 2009; Guillén-Navarro et al., 2013). BSCL2 arises *via* loss-of seipin function, whereas neuronal disorders may be related to accumulation of toxic seipin oligomers (Rao and Goodman, 2021). Mechanistically, seipin localizes to ER-LD contact sites and is crucial for LD formation across the evolutionary tree (Chapman et al., 2019). Seipin is also required for adipogenesis and adipocyte maintenance, and it is an important open question whether the as-yet-unresolved molecular function of seipin at LDs is the culprit for defective adipogenesis.

As there are several excellent recent reviews on this topic (e.g. (Gao et al., 2019; Renne et al., 2020; Rao and Goodman, 2021; Schneiter and Choudhary, 2022), I will focus on most recent insights and emerging themes in the ongoing voyage to uncover the molecular function of seipin, a “mysterious” protein (Agarwal and Garg, 2004).

### 1.1 Seipin-defined domains in LD biogenesis

During LD biogenesis, neutral lipids (NLs, triglycerides, TAG, and sterol esters, SE) accumulate within the ER bilayer, with rising concentrations leading to the formation of nanoscale lenses (Thiam and Ikonen, 2020). Lenses have been predicted by simulations (Khandelia et al., 2010; Zoni et al., 2021a; Kim et al., 2022b) and 40–60 nm-in-size lenses have been observed in yeast by electron microscopy (EM) (Choudhary et al., 2015). Upon further growth lenses bud out to the cytoplasmic side of the ER as nascent LDs, which remain in contact with the ER. It is accepted that virtually all LDs remain in contact with the ER in yeast (Jacquier et al., 2011). In mammalian systems it is often stated that LDs detach from the ER after their formation, but there is no clear evidence supporting this. Instead, LDs likely remain in contact with the ER (Salo et al., 2016), and this connection may only be severed in specialized cases, such as during LD engulfment to degradatory compartments (Schulze et al., 2020) or in milk-secreting epithelial cells of the mammary gland (Monks et al., 2020).

Nascent LD formation is influenced by biophysical factors, including local ER lipid composition, membrane shape and surface tension (Ben M’barek et al., 2017; Choudhary et al., 2018; Chorlay et al., 2019; Santinho et al., 2020; Zoni et al., 2021a). Specific proteins also impact LD assembly, including FIT2, DFCP1, LDAF1, ACSL3, perilipins, ER curvature proteins and the yeast lipin orthologue Pah1 (Adeyo et al., 2011; Kassan et al., 2014; Choudhary et al., 2015; Gao et al., 2017; Li D. et al., 2019; Chung et al., 2019; Chen et al., 2021). An elegant recent study ordered the recruitment of many of these proteins during LD formation in yeast, providing clear evidence for a stepwise assembly of LDs (Choudhary et al., 2020). In this LD assembly cascade, seipin plays a pivotal role in both LD nucleation and controlling subsequent LD growth at ER-LD contacts.

By live cell microscopy in human cells, partially immobilized seipin complexes preclude the recruitment of many LD markers, such as ACSL3, Perilipin-3, Bodipy, LD540 or LiveDrop (Chung et al., 2019; Salo et al., 2019). MCTP2 was also recently shown to mark nascent LD formation sites in the ER (Joshi et al., 2018; 2021), MCTP2-sites appeared more immobile than seipin-defined sites. DFCP1 (Li D. et al., 2019) and FIT2, Reep5 and Rtn4A (Chen et al., 2021) were also recently shown to transiently accumulate at ER sites prior to LiveDrop appearance. As these proteins likely harbor membrane-shaping capabilities, these data further imply that ER curvature is vital for early LDs (Santinho et al., 2020). It will be crucial in the future to investigate the temporal relationship of these players in respect to seipin.

### 1.2 Seipin and phospholipids

**FIGURE 1:**
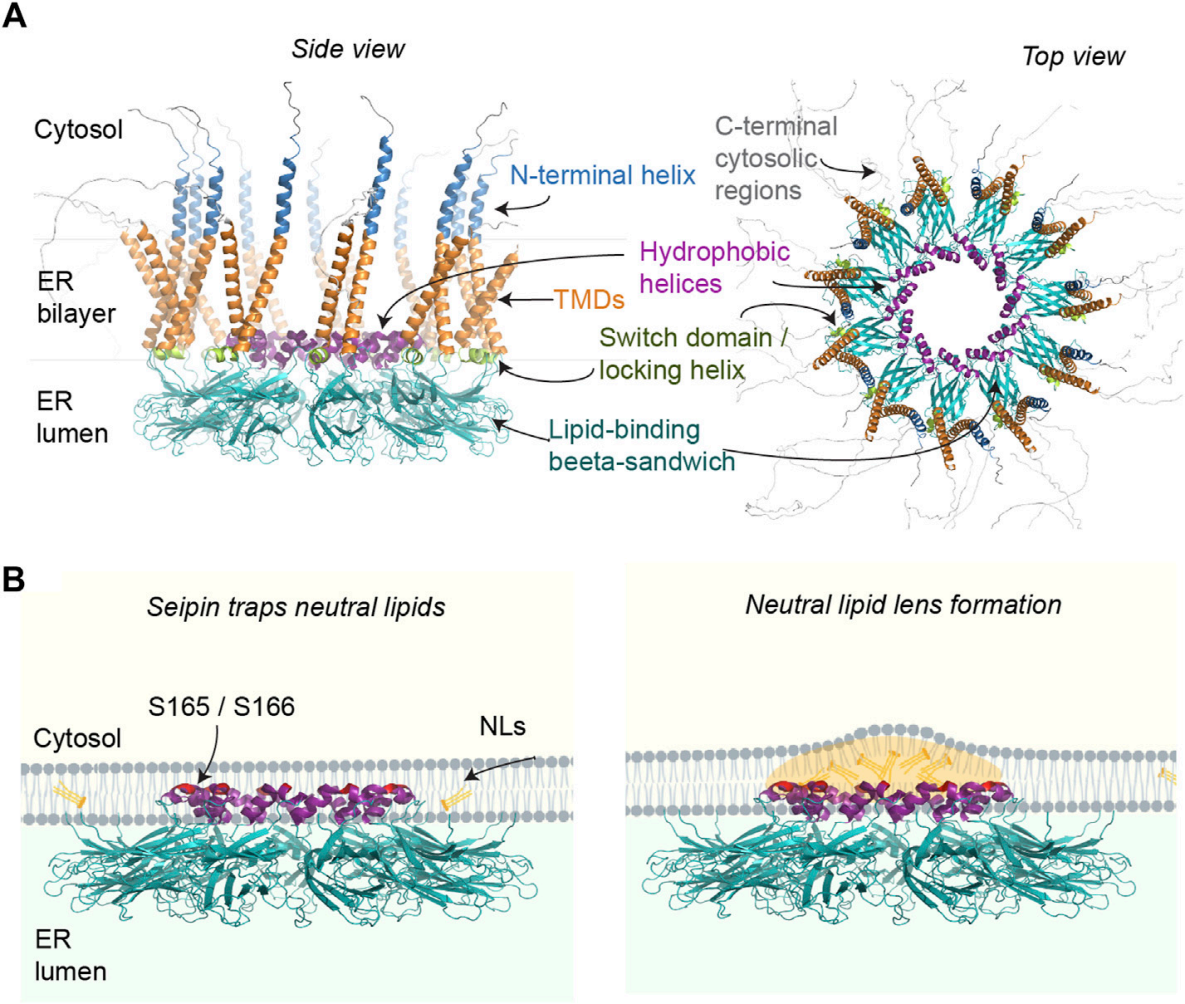
**(A)** Putative structural elements of the human seipin oligomer (isoform 1), predicted using Alphafold Multimer (Evans et al., 2022) and visualized in PyMol. Only the luminal regions containing the hydrophobic helices and lipid-binding beta sandwich of human seipin have been solved by cryo-EM. The rest of the annotations are based on published yeast structures and functional analyses. Alphafold predictions suggest cytosolic N- and C-terminal regions (only partially shown) may be mostly disordered *in vivo*. **(B)** Working model for how seipin may catalyze LD formation by facilitating NL nucleation in the ER. For clarity, only select ER luminal regions of seipin are depicted, with the putative NL-interacting residues highlighted in red.

Seipin homo-oligomers consist of N- and C-terminal cytosolic regions and a conserved luminal domain flanked by two transmembrane domains (TMDs) (Lundin et al., 2006; Binns et al., 2010). The cryo-EM structure of human and *Drosophila* seipin luminal domains indicates that seipin forms ER luminal disks of 11–12 subunits, with a diameter of ∼15 nm (Sui et al., 2018; Yan et al., 2018) (Figure 1A). The outer layer of this ring is composed of closely interacting beta-sandwich folds with structural similarity to lipid-binding domains, binding anionic phospholipids *in vitro* (Yan et al., 2018). These putative phospholipid binding domains are especially interesting in the context that seipin has been implicated in localized control of phosphatidic acid (PA) metabolism (Fei et al., 2011; Sim et al., 2012; 2020; Jiang et al., 2014; Han et al., 2015; Talukder et al., 2015; Wolinski et al., 2015; Pagac et al., 2016; Soltysik et al., 2020). Indeed, one model for seipin function is that it prevents ectopic accumulation of PA in the ER, which could lead to both aberrant LD formation and disturb the adipogenic programme by interfering with the key transcription factor PPAR-gamma (Rao and Goodman, 2021).

Besides PA, a recent study indicated that PI3P, another anionic phospholipid, accumulates in ER foci in seipin KO (Lukmantara et al., 2022). Remarkably, decreasing PI3P was sufficient to partially rescue the LD phenotype of seipin KO. Increased PI3P *via* seipin KO was shown to impair the activity of DFCP1 in facilitating early LD formation. Seipin and DFCP1 have been mechanistically linked previously (Li D. et al., 2019) and although endogenous proteins do not directly interact (Lukmantara et al., 2022) it will be crucial to further decipher their interplay in LD formation. As DFCP1 harbors NTPase activity (Ismail et al., 2022; Nähse et al., 2022) facilitating omegasome constriction (Nähse et al., 2022) it could be directly involved in membrane deformation during LD budding.

### 1.3 Seipin nucleates LDs

Seipin also contains hydrophobic helices (HHs) in the center of the ring, partially embedded in the ER bilayer. Molecular simulations supported by mutational analysis indicate that the HHs may induce nanoscale clustering of TAG molecules (Zoni et al., 2021b; Klug et al., 2021; Prasanna et al., 2021; Kim et al., 2022a; Renne et al., 2022) (Figure 1B **)**. Importantly, seipin induces this TAG lensing at a lower concentration than TAG alone, providing a plausible explanation why LDs form at sites marked by seipin and ER TAG is increased in seipin KO (Gao et al., 2017; Salo et al., 2019; Choudhary et al., 2020).

Biochemically, TAG has been found to co-purify with seipin, but only in the presence of its important interactor LDAF1, a likely homolog of yeast ldo proteins (Eisenberg-Bord et al., 2018; Teixeira et al., 2018; Castro et al., 2019). Indeed, analysis using acute seipin removal with the auxin-inducible degradation system indicated that LDAF1 may serve to protect seipin-TAG lenses (Prasanna et al., 2021). It is plausible that even in TAG-deprived situations minute amounts of TAGs may become stably clustered within the seipin disk, with a high number of seipin-LDAF1 complexes always primed for LD formation. In the absence of LDAF1, LDs are fewer in number but larger (Chung et al., 2019; Chartschenko et al., 2021), which could be a consequence of defectively primed (“TAGless”) seipins needing higher ER TAG concentrations before LD formation can begin.

### 1.4 New structures, new insights

The structure of yeast seipin was recently resolved by cryo-EM, and shows an overall similar fold to its mammalian counterparts, albeit with 10 subunits (Klug et al., 2021; Arlt et al., 2022). In yeast, seipin function is carried out by two interacting proteins, Sei1 and Ldb16, which form a stable complex. However, density could not be resolved for Ldb16, indicating structural flexibility. Nevertheless, the structures of Sei1 and supporting cross-linking analysis revealed that in yeast the HHs important for LD nucleation are likely provided by Ldb16 (Klug et al., 2021).

The structures also resolved seipin TMDs and one of the structures indicated that the TMDs of seipin exist in two separate conformations (Arlt et al., 2022). It was speculated that during LD formation the seipin ring may thus open up *via* a conformational switch of the TMDs as additional TAGs are accommodated. In line with this, a conserved, luminal region near the TMDs, called switch domain or locking helix, appears to be important to control TMD orientation and flexibility (Klug et al., 2021; Arlt et al., 2022). Indeed, simulations indicate that the TMDs and nearby residues are important for seipin function in both attracting TAGs in the ER bilayer (Zoni et al., 2021b) and facilitating the conversion of a flat TAG-lens into a budding LD (Kim et al., 2022a).

### 1.5 Seipin and ER-LD contacts

**FIGURE 2:**
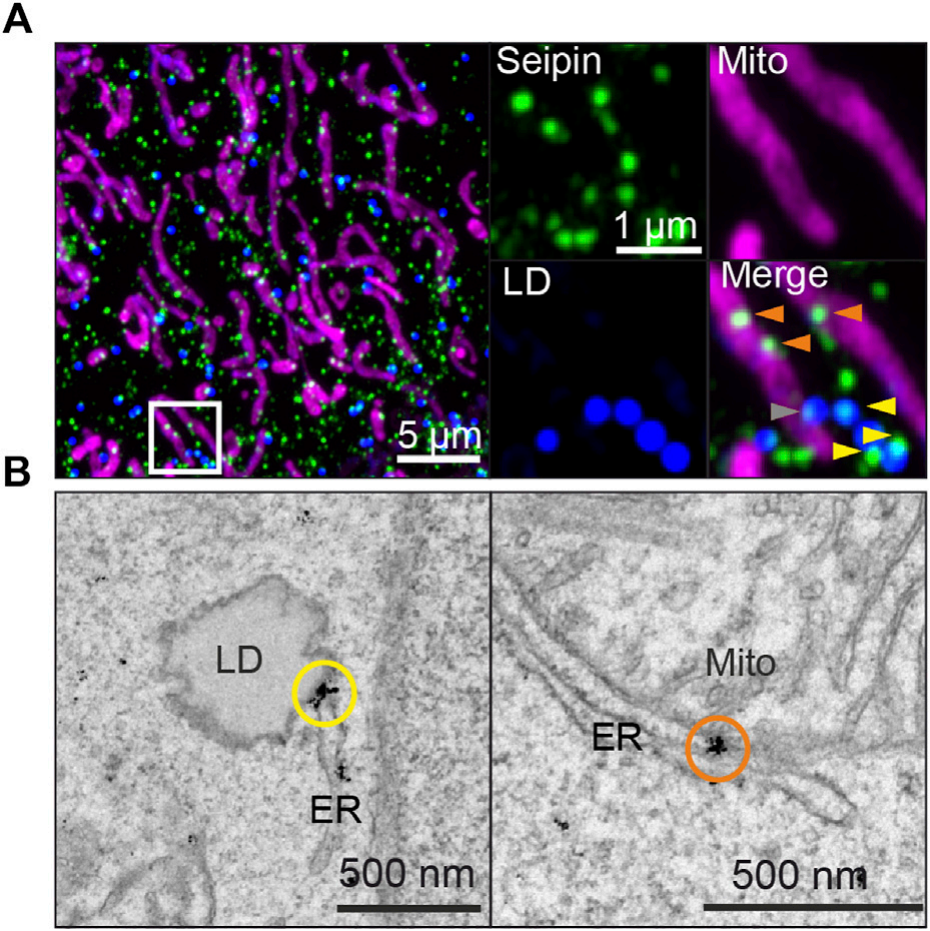
Endogenously tagged seipin localizes to both ER-LD and ER-mitochondria contact sites in human A431 cells, as shown by **(A)** Airyscan microscopy of fixed cells and **(B)** immuno-EM gold labeling. Yellow arrowheads and circles: seipin localization at ER-LD contact sites, orange arrowheads and circles: seipin localization at ER-mitochondria contact sites. Gray arrowheads indicate seipins near both sites simultaneously. For detailed methodology, see (Combot et al., 2022) from wherein image is adapted from.

It is well documented that many seipin foci (likely corresponding to individual seipin oligomers although this has not been formally shown) localize stably to ER-LD contact sites in yeast, *Drosophila, C. elegans* and human cell models (Szymanski et al., 2007; Fei et al., 2008; Salo et al., 2016; Wang et al., 2016; Cao et al., 2019) (Figure 2). In human non-adipocyte cell models, there is typically one seipin-mediated contact per LD, which displays direct membrane continuity, possibly as a consequence of LD budding taking place through the seipin disk (Salo et al., 2020). At ER-LD contacts, seipin appears to be required for continuous LD growth *via* the ER-LD contact (Salo et al., 2019) and protein trafficking between the ER and LDs (Grippa et al., 2015; Salo et al., 2016; Cottier and Schneiter, 2022). The aforementioned TAG-attracting propensity of seipin may be important for this (Zoni et al., 2021b; Prasanna et al., 2021). Seipin may physically stabilize the contact site, which is supported by the notion that by electron tomography seipin-mediated ∼15 nm ER-LD necks display uniform membrane architecture and size (Salo et al., 2019). The TMDs of seipin, likely exhibiting a degree of flexibility, may also effect phospholipid and/or protein diffusion at the ER-LD neck. Recent *in vitro* studies in protein-free systems have begun to shed light on the unique properties of ER-LD membrane continuities (Chorlay et al., 2021; Puza et al., 2022), paving the way to understand the contribution of protein factors such as seipin in ER-LD cargo exchange.

In addition to seipin-mediated ER-LD contact sites, there are also other types of ER-LD contacts, including wider ER-LD membrane proximities resembling canonical membrane contact sites (Salo and Ikonen, 2019; Bohnert, 2020). These may be regulated by ER-LD tethering factors such as Rab18, Snx14, Vps13 and MOSPD2 (Kumar et al., 2018; Xu et al., 2018; Datta et al., 2019; Zouiouich et al., 2022) although the division of labor between such contact sites and direct membrane continuities is unclear.

Recently, in a *tour de force* study *Drosophila* cells*,* a specific set of membrane-fusion machinery was proposed to generate seipin-independent ER-LD membrane bridges to enable specialized protein cargo, such as GPAT4, to traffic to a subset of LDs upon their maturation (Song et al., 2022). Using FRAP, GPAT4 was found to traffic to LDs *via* the ER at sites not directly harboring a seipin complex (Song et al., 2022). However, the resolution of light microscopy in the crowded ER-LD network makes interpretation of such data challenging. An alternative possibility is that the described protein machinery acts on matured LDs and directly alters their surface properties, such as membrane tension (Gong et al., 2011; Thiam et al., 2013), thus allowing specific protein cargo to travel to the LD *via* the existing seipin-mediated ER-LD neck. In any case, it will be interesting to compare the membrane architecture and nanoscale dimensions of these seipin-dependent and -independent contact sites and how this correlates with their functionality.

### 1.6 Seipin and sterol esters

To date, most reports of seipin in LD formation have focused on TAG-LDs. However, recent work has begun to shed light on the role of seipin specifically in SE-LDs. SE levels were unaltered in BSCL2 patient lymphoid cells (Boutet et al., 2009), decreased in seipin KO A431 cells (Salo et al., 2016) and slightly increased in seipin KO yeast (Wang et al., 2014; Grippa et al., 2015). As seipin has been documented to reside at ER-LD contacts of SE-LDs in both yeast and human cells (Santinho et al., 2020; Molenaar et al., 2021), seipin may also play a role in SE-LD formation. However, the assembly of SE-LDs may be quite different to TAG-LDs due to different physical chemistry (Thiam and Ikonen, 2020). Indeed, in contrast to TAG-LDs, seipin could not determine SE-LD biogenesis site in yeast (Molenaar et al., 2021) suggesting seipin-independent pathways for SE-LD formation. Seipin could also not determine the biogenesis site for retinyl ester-LDs, although it did facilitate their packaging into pre-existing TAG-LDs (Molenaar et al., 2021).

In a recent study, careful quantification of LD sizes in yeast seipin mutants engineered to produce SE- or retinyl ester -only LDs revealed marked alterations in their sizes (Renne et al., 2022). Simulations also showed that the HHs in the seipin ring could cluster SEs similarly as TAGs, suggesting seipin could facilitate SE-LD nucleation (Renne et al., 2022). These data suggest seipin can act as a universal NL-clustering machine in the ER, although some of the observed differences may also be caused by defective ER-LD lipid flux rather than defective initial LD formation. Very recently seipin was also shown to be essential for SE-LD maintenance and formation *in vivo,* in steroidogenic tissues of mice (Shen et al., 2022).

Interestingly, emerging work suggests that LDs can undergo phase transition, with liquid crystalline lattices detected by *in situ* cryo-electron tomography and linked to high SE/TAG ratios (Czabany et al., 2008; Mahamid et al., 2019; Shimobayashi and Ohsaki, 2019). Driven by lipolysis of TAGs or other metabolic cues (Suzuki et al., 2012; Mahamid et al., 2019; Rogers et al., 2022), rising SE concentrations thus lead to the formation of lattices at the periphery of LDs, whilst LD cores remain amorphous with TAGs. How such lattices may impact the nanoscale architecture of seipin-mediated ER-LD contact sites remains to be investigated, but proteomic changes in SE vs. TAG LDs (Hsieh et al., 2012; Khor et al., 2014; Rogers et al., 2022) could be related to altered functionality of seipin-mediated ER-LD necks.

### 1.7 Seipin at mitochondria-ER contact sites

In addition to its role in LD assembly and PA metabolism, several studies have linked seipin function to calcium fluxes. Seipin was found to physically interact with the ER calcium pump SERCA in *Drosophila,* acting as a positive SERCA regulator (Bi et al., 2014). In seipin KO flies, ER calcium was reduced and fat body lipogenesis defects could be partially rescued by disturbing ER-to-cytosol calcium efflux. In a follow-up study, it was found that the defective ER calcium stores led to decreased mitochondrial calcium and concomitant defects in TCA cycle and mitochondrial function (Ding et al., 2018). Indeed, several studies have since uncovered altered calcium homeostasis in seipin knockdown systems (Li Q. et al., 2019; Wu et al., 2021).

We recently investigated seipin links to mitochondria and calcium fluxes using human and mouse cells as well as inducibly seipin depleted mice. We found that within hours of acute seipin removal, calcium flux to mitochondria was reduced, whilst ER calcium stores remained initially intact (Combot et al., 2022). Reduced mitochondrial calcium flux was accompanied by mitochondrial defects in multiple cell types including BSCL2 patient cells. Mechanistically, a subset of seipins were found to localize at ER-mitochondria contact sites (MsAMs) (Figure 2) in a nutritionally regulated manner, in close proximity to calcium regulators SERCA and IP3R, suggesting seipin may be important in stabilizing MAMs for calcium flux into mitochondria.

Another recent study also reported that a subset of seipin localized to MAMs in human cells (Guyard et al., 2022). They found that an Orp5/Orp8 complex is important for LD formation and localizes to newly described ER-MAM-LD tripartite contact sites, wherein LDs were proposed to emerge. Importantly, Orp5 was necessary to recruit seipin to MAMs, which were also found to be enriched in PA. Interestingly, whilst (Guyard et al., 2022) linked MAM-seipins to LD formation, (Combot et al., 2022), found MAM-seipin localization to decrease in lipogenic conditions and increase in starvation. These discrepancies could be related to differences between cell-types. Altogether, nutritional status of the cell may effect MAM lipid environment, leading to seipin recruitment *via* Orp5, wherein seipin could be important for stabilizing calcium-regulating ER subdomains.

These studies highlight the emerging concept that seipin may be involved in multiple ER contact sites and/or organelle assembly platforms (LDs and mitochondria in mammals and LDs and nascent peroxisomes in yeast (Joshi et al., 2018; Wang et al., 2018)). Interestingly, two seipin isoforms in *Arabidopsis* were recently shown to physically interact with VAP during LD formation (Greer et al., 2020). Whilst mammalian seipin contains no obvious VAP-interacting FFAT motif, pulldowns of endogenously tagged mouse seipin did contain VAPA (Combot et al., 2022), prompting further study. The dynamic mobility of most MAM-associated seipins (Combot et al., 2022) appears similar to the dynamic VAPB MAM domains recently described by single molecule imaging (Obara et al., 2022).

### 1.8 Future outlook

At present, the two main, mutually non-exclusive hypothesis of seipin function (control of phospholipid metabolism and as a LD nucleator) are both supported by strong experimental clues. There appear to be at least three, partially overlapping populations of seipins: ER, ER-LD and ER-mitochondrial seipins. These may exert different functions due to altered conformations or binding partners. For further cataloguing of seipin subcellular behavior and localization, it would be important to investigate endogenous-level seipin, since overexpression may alter or mask its true localization (Lak et al., 2021). Furthermore, care should be exercised when assessing seipin LD phenotypes; LD markers of sufficient sensitivity are required to fully characterize the typical seipin KO phenotype of tiny and supersized LDs (Salo et al., 2020).

In regards to seipin-TAG lenses, direct observations of them have not been reported and the previously reported lenses in yeast cells seem too large to be contained within the seipin disk (Choudhary et al., 2015). Going forward, reconstitution of seipin in TAG-enriched model membranes would help to tackle its role in LDs, aided by recent advances in mimicking LD nucleation in protein-free systems (Santinho et al., 2020; Hegaard et al., 2022). High resolution *in situ* cryo-EM could be another avenue to directly visualize seipin-mediated early LDs. Further structural work on seipin in complex with its myriad interactors will also be crucial to decipher the function of seipin at different membrane contact sites.
